# Personalized Combination of a Ketogenic Diet and Low-Dose Semaglutide for Cardiometabolic Health: A Retrospective Case Series

**DOI:** 10.3390/jpm16060313

**Published:** 2026-06-12

**Authors:** Genevieve Parker, Madeline D. Morris, Jeter R. Heggie, Ella F. Cooper-Leavitt, Cameron J. Clark, Asher P. Reynolds, Holly A. Smith, Carlie P. Wendel, William J. Jensen, Tyson J. Morris, Paul R. Reynolds, Benjamin T. Bikman

**Affiliations:** 1 Department of Cell Biology and Physiology, Brigham Young University, Provo, UT 84604, USA; 2 Summit Family Health-Metabolic Clinic, Meridian, ID 83642, USA; 3 School of Medicine, Creighton University, Phoenix, AZ 85012, USA

**Keywords:** ketogenic diet, semaglutide, GLP-1 receptor agonist, insulin resistance, body composition, lean mass preservation, visceral fat, case series, personalized medicine, individualized therapy

## Abstract

**Background/Objectives:** Glucagon-like peptide-1 receptor agonists (GLP-1 RAs), particularly semaglutide, have demonstrated efficacy for weight loss in obesity; however, up to 40% of weight lost may derive from lean body mass. The ketogenic diet independently improves insulin sensitivity and promotes fat oxidation while preserving lean tissue. This study aimed to describe changes in body composition, insulin sensitivity, and cardiometabolic markers in patients who followed a personalized ketogenic dietary protocol while receiving low-dose semaglutide over a 6-month insulin resistance reversal program. **Methods:** Seven analyzed adults (six female, one male) with overweight or obesity (baseline BMI 25.6–47.2 kg/m^2^) participated in a clinician-supervised 6-month program combining a whole-food ketogenic diet with semaglutide (≤1.0 mg/week). Body composition and fasting metabolic markers were assessed at 1, 3, and 6 months. **Results:** Mean total weight loss was 21.9 kg, of which a mean of 92% was attributable to BIA-estimated fat mass. Skeletal muscle mass was largely preserved as measured by BIA (mean loss 1.2 kg), and one patient gained lean tissue. Fasting insulin declined by a mean of 15.6 µIU/mL. Visceral fat decreased by a mean of 37.0%. Six of seven patients showed reductions in high-sensitivity C-reactive protein. Triglycerides decreased in six of seven patients, and HDL cholesterol increased in all seven. LDL cholesterol responses were heterogeneous. **Conclusions:** In this small, uncontrolled case series, combining a ketogenic diet with low-dose semaglutide was associated with substantial fat loss, apparent preservation of lean mass as measured by BIA, and improvements in insulin sensitivity and cardiometabolic markers. Because the semaglutide dose and dietary protocol were individualized to each patient’s response, the program illustrates a personalized approach to insulin resistance. These preliminary findings are hypothesis-generating and warrant confirmation in controlled prospective studies.

## 1. Introduction

Insulin resistance is increasingly recognized as a central driver of metabolic disease, preceding the development of obesity, type 2 diabetes, cardiovascular disease, and related cardiometabolic complications [1]. Elevated fasting insulin often precedes hyperglycemia and serves as an early marker of metabolic dysfunction, even when glucose values remain within normal ranges [2]. Two therapeutic approaches that have been widely studied in the context of insulin resistance are glucagon-like peptide-1 receptor agonists (GLP-1 RAs) and the ketogenic diet.

Semaglutide, a GLP-1 RA, enhances glucose-dependent insulin secretion, suppresses glucagon release, slows gastric emptying, and reduces appetite [3]. In the STEP-1 trial, patients taking semaglutide lost a mean of 14.9% of body weight, with over one-third exceeding 20% weight loss [4]. However, a significant limitation is the associated loss of lean body mass, which may account for 25–40% of total weight lost [4,5]. This is of particular concern for postmenopausal women and older adults who are already at elevated risk for sarcopenia [6].

The ketogenic diet induces nutritional ketosis by sufficiently restricting carbohydrate intake, prompting the liver to convert dietary and stored fat into ketone bodies. This metabolic shift lowers circulating insulin levels and promotes substantial fat loss, including visceral fat reduction, while preserving lean mass when protein intake is adequate [7]. Beyond fat loss, the ketogenic diet has been shown to reduce triglycerides, improve glucose and lipid profiles, and lower systemic inflammation [8,9].

The pharmacological appetite suppression of semaglutide, combined with the metabolic shift toward fat oxidation promoted by the ketogenic diet, may act through complementary mechanisms relevant to insulin sensitivity, inflammation, and visceral fat. The high protein intake and ketone-mediated anti-catabolic signaling characteristic of the ketogenic diet have also been proposed as a potential means of attenuating the lean mass loss associated with GLP-1 RA therapy. A recent randomized pilot trial has evaluated semaglutide combined with a very-low-calorie diet in individuals with type 2 diabetes, reporting favorable effects on body weight, fat mass, insulin resistance, and beta-cell function [10]. The present report differs in that it examines a ketogenic dietary framework rather than a very-low-calorie diet and focuses on a broader population with insulin resistance rather than established type 2 diabetes. The purpose of this report is to describe the outcomes of seven patients who combined a ketogenic diet with low-dose semaglutide as part of a 6-month insulin resistance reversal program. Importantly, each patient’s semaglutide dose was individually titrated to the lowest effective level, and the dietary protocol was adapted to individual tolerance and metabolic response—an approach consistent with the principles of personalized medicine in which therapeutic interventions are tailored to the individual patient’s metabolic phenotype rather than applied as a uniform regimen.

## 2. Materials and Methods

### 2.1. Study Design and Participants

This is a retrospective case series of eight patients enrolled in a clinician-supervised, 6-month insulin resistance reversal program. All patients who completed the full 6-month program and had complete InBody and laboratory data at all three time points were included in this analysis; one completer was excluded as a protocol deviation (discontinuation of semaglutide and transition to phentermine), yielding seven analyzed participants. Throughout the program, eight individualized consultations were provided, and semaglutide was prescribed at the lowest effective dose. Most participants began at 0.25 mg/week and titrated to 0.5 mg/week after one to two weeks. Most participants were maintained at 0.5 mg/week; one down-titrated to 0.25 mg/week, and one was maintained at 1.0 mg/week. No patient exceeded 1.0 mg/week, and per-patient trajectories are shown in Table 1. Both the semaglutide dose and the dietary plan were individualized to each patient’s tolerance, glucose and ketone response, and clinical progress, consistent with a personalized-medicine approach rather than a fixed protocol.

### 2.2. Dietary Intervention

Patients were instructed to follow a ketogenic diet based on three core principles: (1) control carbohydrates by consuming only vegetables that grow above the ground; (2) prioritize protein by including a whole-food, animal-based protein source at every meal; and (3) include dietary fat without intentionally increasing fat intake. To monitor ketosis, patients wore a continuous glucose monitor and aimed to maintain blood glucose below 90 mg/dL while tracking urine ketones with a target above 0.5 mmol/L. Dietary guidance was provided by the supervising clinician at each of the eight individualized consultations, including instruction on meal planning, appropriate protein portions, and eligible food choices. Protein intake was not formally quantified by food diary or dietary recall. Exercise or resistance training was encouraged but was not standardized or systematically tracked as part of the protocol. Intermittent fasting was not a required component of the program, though some patients incorporated it independently. Dietary adherence was monitored indirectly through continuous glucose monitoring data and urine ketone measurements; formal adherence instruments were not employed. Quantitative continuous glucose monitoring trends and urine ketone logs were not systematically retained, so nutritional ketosis was not objectively verified for individual participants.

### 2.3. Outcome Measures

InBody scans were performed at the 1-, 3-, and 6-month time points to assess total body weight (kg), body mass index (BMI, kg/m^2^), skeletal muscle mass (SMM, kg), and visceral fat (reported as the InBody visceral fat level, a unitless index derived from segmental bioelectrical impedance and expressed here as percentage change from baseline). Because bioelectrical impedance estimates of fat and skeletal muscle mass are sensitive to hydration and glycogen shifts—pronounced during ketogenic adaptation—these values are reported as BIA-estimated and interpreted with corresponding caution. Fasting bloodwork was collected on the same schedule and included fasting glucose (mg/dL), fasting insulin (µIU/mL), hemoglobin A1c (%), alanine aminotransferase (ALT, U/L), aspartate aminotransferase (AST, U/L), high-sensitivity C-reactive protein (hsCRP, mg/L), vitamin D (ng/mL), and a full lipid panel comprising total cholesterol, LDL, HDL, VLDL, and triglycerides (all mg/dL). Although assessments were conducted at months 1, 3, and 6, this report focuses on baseline-to-month-6 comparisons because month-1 and month-3 data were incomplete across all participants and the primary clinical question concerned cumulative 6-month outcomes; the intermediate time points are noted as a limitation.

### 2.4. Ethical Considerations

The retrospective analysis used de-identified data. Informed consent for the research analysis was not required. This study was conducted in accordance with the Declaration of Helsinki. The Institutional Review Board of Brigham Young University determined this retrospective analysis to be exempt from formal review pursuant to applicable federal guidelines for secondary research using de-identified data (45 CFR 46.104(d)(4)), as the study involved no direct patient contact or intervention beyond routine clinical care, and all data were de-identified prior to analysis.

### 2.5. Use of Artificial Intelligence

During the preparation of this manuscript, the authors used Claude and Grok to assist in identifying and screening potentially relevant published literature for citation. All sources surfaced by these tools were independently retrieved, read, and verified by the authors for accuracy and relevance before inclusion. The authors reviewed and edited all content and take full responsibility for the content of the publication.

### 2.6. Statistical Analysis

Given the small sample, analyses are descriptive; data are summarized as mean (range) and median (interquartile range). Exploratory Wilcoxon signed-rank tests of within-participant change were computed for principal outcomes and interpreted as hypothesis-generating only, unadjusted for multiple comparisons (with *n* = 7 the minimum attainable two-sided *p* is 0.016). On this exploratory basis, body weight, fat mass, visceral fat, fasting insulin, and HDL cholesterol changed at *p* = 0.016 and triglycerides at *p* = 0.031, whereas skeletal muscle mass, HbA1c, hsCRP, and LDL cholesterol were not statistically significant. The study is reported in accordance with the STROBE and CARE reporting guidelines.

## 3. Results

### 3.1. Patient Characteristics

Seven patients (six female, one male) were analyzed (see Methods). Baseline BMI ranged from 25.6 to 47.2 kg/m^2^ (mean 38.7). Participant ages were 42, 63, 40, 53, 40, 37, and 49 years (mean 46, range 37–63). All participants were of White/European background; among the six women, five were premenopausal and one postmenopausal. Comorbidities included type 2 diabetes (*n* = 1), hyperlipidemia (*n* = 2), and hypertension (*n* = 1). Baseline concomitant medications were more extensive than previously tabulated and are detailed in Table 1; medication changes during the program are summarized in Table 2. Two patients had received tirzepatide before enrollment (Patient 4 for approximately 4 months, active until enrollment; Patient 6 for approximately 2 months). Patient demographics and baseline characteristics are presented in Table 1.

### 3.2. Body Composition Outcomes

Over the 6-month intervention, mean total weight loss was 21.9 kg (range 12.0–29.4). A mean of 92% of total weight lost was attributable to BIA-estimated fat mass (range 84–100%). Skeletal muscle mass was largely preserved, with a mean loss of only 1.2 kg; notably, Patient 4 gained 1.1 kg of lean tissue. Mean BMI decreased by 7.2 points (range 2.3–11.8). Visceral fat decreased by a mean of 37.0% (range 15–67%). Complete body composition outcomes are presented in Table 3. Baseline and 6-month BIA-estimated values are provided in Table 4.

### 3.3. Metabolic and Inflammatory Markers

Fasting insulin declined in all patients, with a mean reduction of 15.6 µIU/mL (median −10.4), reflecting substantial improvement in insulin sensitivity. Hemoglobin A1c improved in four patients, remained stable in two, and increased marginally in one (+0.3%). Patient 2, the only participant with baseline type 2 diabetes (A1c 9.8%), demonstrated the most dramatic glycemic improvement, reducing to 6.7% (interpretation confounded by concurrent discontinuation of glipizide and dapagliflozin; see Limitations). Six of seven patients showed reductions in hsCRP, indicating decreased systemic inflammation; Patient 5 achieved a 75% reduction (36.77 to 9.4 mg/L). Patient 2 exhibited a rise in hsCRP (10.93 to 13.94 mg/L), possibly reflecting persistent low-grade inflammation associated with ongoing obesity. Liver enzymes generally improved or remained stable.

### 3.4. Lipid Profiles

Triglycerides decreased in six of seven patients, with a mean reduction of 78 mg/dL. HDL cholesterol increased in all seven patients (mean +7.9 mg/dL). LDL cholesterol responses were heterogeneous: five of seven patients showed increases ranging from +10 to +73 mg/dL, while two showed decreases. VLDL decreased in three patients, remained stable in two, increased modestly in one, and was unavailable in one. Complete metabolic and lipid data are presented in Table 5. Individual responses varied notably across glycemic, inflammatory, and lipid measures, underscoring the value of evaluating outcomes at the individual rather than the group level.

## 4. Discussion

The findings of this case series are best understood as preliminary, hypothesis-generating observations from an uncontrolled retrospective analysis. The outcomes are consistent with a speculative clinical paradigm worthy of prospective testing: the time-limited, low-dose use of semaglutide as a dietary transition aid to facilitate adoption of a ketogenic diet, rather than as a chronic weight management drug. Each outcome observed is mechanistically consistent with the ketogenic diet as the primary therapeutic agent, with semaglutide serving a targeted, time-limited behavioral function during the early transition period. However, without comparator arms receiving semaglutide alone or the ketogenic diet alone, no causal or synergistic inference can be drawn from these data.

The most visually striking finding was the quality of weight loss: a mean of 92% of total weight lost was attributable to BIA-estimated fat mass, compared with the 60–75% typically reported in semaglutide monotherapy trials [4,5]. These figures must be interpreted cautiously, given differences in patient population, semaglutide dose, dietary protein intake, measurement methodology, and the absence of a comparator arm, and the monotherapy values are offered as context rather than as a direct comparison. Nonetheless, only 8% of the weight lost in this cohort was BIA-estimated lean mass, and one patient gained skeletal muscle mass during active weight reduction. BIA-derived body composition estimates are sensitive to hydration status, which changes substantially during ketogenic adaptation as glycogen-bound water is mobilized; the apparent lean mass preservation figures should therefore be interpreted with this methodological caveat in mind. Semaglutide monotherapy, particularly at high doses, produces weight loss primarily through caloric restriction without directing the source of that deficit. Lean mass losses of 25–40% are therefore a predictable consequence of unconstrained energy reduction in the absence of dietary protein prioritization [4,5,6].

The ketogenic diet, by contrast, emphasized protein during counseling, although protein intake was not quantified; ketone-mediated anti-catabolic signaling has been proposed as a parallel contributor. The ketone body βHB may suppress muscle protein breakdown [11], reduces reliance on gluconeogenesis from amino acids [12], and—as we have demonstrated previously across cell, rodent, and human models—increases mitochondrial uncoupling in adipose tissue, increasing energy expenditure in adipose preferentially [13]. These mechanisms were not assessed in the present cohort and are presented as hypotheses for future testing. A recent randomized controlled trial reported that exogenous βHB supplementation reduced fat mass while preserving lean tissue during caloric restriction [14]. Taken together, these mechanisms provide a plausible explanation for the favorable body composition pattern observed in this cohort. However, the small sample size, absence of a comparator arm, and reliance on BIA rather than a criterion reference method preclude definitive conclusions about the degree of lean mass preservation attributable to the combination.

All patients demonstrated substantial reductions in fasting insulin, with a substantial mean decline (−15.6 μIU/mL)—reflecting a meaningful recovery of insulin sensitivity. Elevated fasting insulin in the absence of frank hyperglycemia is increasingly recognized as the earliest detectable marker of metabolic dysfunction, preceding overt glucose dysregulation by years to decades [2]. The mechanisms through which the ketogenic diet restores insulin sensitivity operate at both the systemic and cellular levels. Systemic carbohydrate restriction eliminates postprandial glucose excursions and the chronic hyperinsulinemia they provoke. Within cells, βHB enhances mitochondrial efficiency and reduces oxidative stress and inflammation—two interlocking mechanisms central to impaired insulin signaling [1,13,15]. Semaglutide contributes complementary mechanisms by helping reduce cravings for refined carbohydrates, thus lowering the need for insulin secretion over time [16]. The convergence of dietary, ketone-mediated, and pharmacological mechanisms targeting insulin resistance from distinct angles likely explains the depth of insulin sensitivity improvement observed in this cohort.

The mean visceral fat reduction of 37.0% observed across six months in this cohort may be contextualized against the 27.4% reduction reported over 68 weeks in a semaglutide monotherapy subpopulation in the STEP 1 body composition analysis [17], noting that the populations, doses, durations, and methods are not directly comparable; the difference in observation period further limits this comparison. These monotherapy figures are presented only as contextual reference points, not as a performance comparison, and should be interpreted cautiously given the differences in study design, sample size, measurement methods, and absence of a concurrent control group. Nonetheless, the magnitude of visceral fat reduction is noteworthy because visceral adiposity is the adipose depot most directly linked to cardiometabolic risk, driving insulin resistance through increased portal delivery of free fatty acids to the liver, promotion of hepatic gluconeogenesis, and constitutive secretion of pro-inflammatory cytokines including interleukin-6 and tumor necrosis factor-alpha [18]. The reductions in hsCRP observed in six of seven patients are consistent with this mechanism: as visceral fat volume declines, the inflammatory tone it sustains diminishes in parallel. The carbohydrate restriction central to the ketogenic diet appears to be particularly effective at targeting visceral fat, likely because visceral adipocytes exhibit relatively greater catecholamine-stimulated lipolysis and reduced sensitivity to insulin’s antilipolytic effect compared to subcutaneous adipocytes, making them disproportionately responsive to the low-insulin environment created by carbohydrate restriction [8].

The lipid profile changes reinforce this interpretation: triglycerides declined in six of seven patients, and HDL cholesterol increased universally, a pattern that precisely mirrors the metabolic consequences of reduced de novo lipogenesis and improved lipoprotein clearance under low-insulin conditions [19,20]. The variable LDL responses observed—including transient elevations in some patients—are consistent with published ketogenic diet literature and may reflect increased flux of mobilized fatty acids through lipoprotein metabolism during rapid fat loss rather than a worsening atherogenic profile [21]. The triglyceride-to-HDL ratio, which has emerged as a more informative predictor of insulin resistance and atherogenic dyslipidemia than LDL concentration alone, improved in the majority of patients [22]. Nevertheless, LDL elevations in several patients—including those with pre-existing hyperlipidemia—warrant clinical attention and ongoing monitoring. The long-term cardiovascular significance of these changes is uncertain, and clinicians should not dismiss LDL increases solely on the basis of favorable triglyceride and HDL trends. Individual lipid responses should inform clinical management decisions, particularly in patients at elevated baseline cardiovascular risk.

These observations also carry implications for individualized, or personalized, management. Because both the semaglutide dose and the ketogenic protocol were titrated to each patient’s tolerance and metabolic response rather than applied as a fixed regimen, the program functioned as a personalized intervention in which therapeutic intensity was matched to the individual. The heterogeneity of response reinforces this framing: the single participant with type 2 diabetes showed the largest glycemic improvement, lipid responses diverged (LDL rose in five of seven participants while triglycerides and HDL improved broadly), and lean-mass preservation is of particular concern for peri- and postmenopausal women at elevated risk of sarcopenia. Such variation argues for individualized monitoring—for example, apolipoprotein B or lipoprotein particle assessment in patients with rising LDL—and for tailoring the dose and duration of semaglutide to the minimum required, consistent with a personalized-medicine approach to insulin resistance rather than a uniform protocol.

Taken together, these outcomes raise a question for future study regarding how semaglutide is positioned in clinical practice. The dominant paradigm positions GLP-1 receptor agonists as chronic weight management drugs requiring indefinite use—an assumption supported by the well-documented pattern of weight regain following discontinuation [23]. However, this paradigm may conflate the drug’s pharmacological effects with its appropriate clinical role. Semaglutide exerts robust suppression of appetite and cravings for sweet and energy-dense foods through direct activation of GLP-1 receptors in hypothalamic and hindbrain reward pathways [16]. Importantly, however, these craving-suppressing effects appear to wane over time: in the STEP 5 trial, improvements in craving for sweet foods were statistically significant at weeks 20 and 52 but were no longer significant at week 104, suggesting that semaglutide’s most clinically valuable behavioral effect—reducing the pull of carbohydrate-dense foods—wanes as the body reaches a new metabolic homeostasis during the weight maintenance phase.

We propose, as a hypothesis for future investigation, that short-course low-dose semaglutide may function most effectively as a time-limited dietary transition aid rather than as a chronic weight management drug and that this approach warrants prospective evaluation in controlled trials. The present uncontrolled case series cannot support this claim but provides an observational rationale for such a study.

Two patients in this cohort had previously received tirzepatide, a dual GIP/GLP-1 receptor agonist, prior to enrollment. Their inclusion may have introduced some heterogeneity in baseline weight-loss responsiveness, as prior GLP-1-based therapy can alter receptor sensitivity and baseline metabolic state. This factor cannot be controlled for in the current analysis and should be noted as a potential source of confounding when interpreting these outcomes.

## 5. Limitations

Several limitations should be considered when interpreting these findings. This was a retrospective observational analysis of a small number of participants, and observed changes cannot be causally attributed to the intervention in the absence of a randomized control group. The absence of a semaglutide-alone or ketogenic-diet-alone comparator arm precludes any determination of additive or interactive effects between the two interventions; the term synergy cannot be supported by this design. Ketogenic diet adherence was monitored indirectly through continuous glucose monitoring data and urine ketone measurements; direct nutritional data such as food diaries or dietary recall were not collected, and it is therefore possible that not all patients achieved or sustained nutritional ketosis throughout the full program. Ketone levels and continuous glucose monitoring data were not consistently collected, limiting insight into participants’ precise metabolic states. No quantitative dietary recall data, CGM trend summaries, or urinary ketone logs were systematically retained; this absence is acknowledged as a substantive limitation, and no mechanistic conclusions about the degree of ketosis achieved can be drawn from these data. Additional confounding factors, including variations in exercise habits, intermittent fasting practices, differences in the quality of individualized coaching, and concomitant medications—which were not held constant and included discontinuation of other glucose-lowering agents (glipizide, dapagliflozin, metformin) and changes to lipid-lowering therapy (atorvastatin reduction; ongoing rosuvastatin and ezetimibe), summarized per patient in Table 2—may have influenced outcomes. The small sample precluded age-stratified analysis. Finally, long-term follow-up remains limited, and the durability of these improvements beyond the 6-month program is unknown.

Body composition was assessed using InBody bioelectrical impedance analysis, which estimates skeletal muscle mass based in part on intracellular water content. During ketogenic dietary adaptation, glycogen depletion and associated reductions in intracellular water may cause BIA to underestimate the true reduction in lean tissue, potentially inflating apparent lean mass preservation. Accordingly, the lean mass retention figures reported here (i.e., the finding that 92% of weight lost was fat mass) should be interpreted with this methodological caveat in mind, and any conclusion regarding lean-mass preservation should be regarded as preliminary until confirmed with a criterion reference method such as dual-energy X-ray absorptiometry (DEXA) in a prospective study.

## 6. Conclusions

This uncontrolled case series presents preliminary, hypothesis-generating observations that combining a ketogenic diet with low-dose semaglutide may produce favorable outcomes across multiple domains of metabolic health, including substantial fat loss with apparent preservation of lean body mass as measured by BIA, improved insulin sensitivity, reduced visceral adiposity, and favorable changes in inflammatory and lipid markers. The apparent lean mass preservation observed in this cohort is of particular clinical interest, given the well-documented concern over sarcopenia associated with GLP-1 RA monotherapy, though confirmation with a criterion reference method in a prospective study is needed. These findings provide an observational rationale for prospective, controlled trials to investigate the potential additive or complementary effects of dietary and pharmacological interventions for the treatment of insulin resistance and obesity. More broadly, the individualized, response-guided combination of diet and low-dose pharmacotherapy described here exemplifies the personalized-medicine approach to insulin resistance that such trials should aim to refine.

## Figures and Tables

**Table 1:** Baseline patient demographics and clinical characteristics.

Patient	Sex	Baseline BMI (kg/m^2^)	Baseline Weight (kg)	Key Comorbidities	Semaglutide Dose (mg/wk)	Concomitant Medications
1	F	38.4	95.2	Hyperlipidemia	0.5 → 0.25	Metformin ER 500 mg; Levothyroxine 75 mcg; Lisdexamfetamine 40 mg
2	M	44.1	143.4	T2DM, HTN, Obesity	0.5	Glipizide ER 10 mg; Dapagliflozin 10 mg; Lisinopril 10 mg; Atorvastatin 40 mg
3	F	41.4	109.4	None reported	0.5	Sertraline 100 mg; Bupropion; Letrozole; Cariprazine; Omeprazole
4	F	25.6	74.2	None reported	0.5	Tirzepatide 5 mg (to enrollment); Metformin; Rosuvastatin; Ezetimibe; Duloxetine; Propranolol; Levothyroxine
5	F	47.2	117	None reported	0.5	None reported
6	F	35.2	87.1	Hyperlipidemia, prior cholecystectomy	1.0	Prior tirzepatide (~2 mo); Amitriptyline
7	F	39.0	106.6	None reported	0.5	Metformin; Omeprazole

**Table 2:** Medication changes during the 6-month program, ascertained from available clinical reconciliation records.

Patient	Medication Changes During the Program
1	Not documented (visit-level reconciliation unavailable)
2	Glipizide and dapagliflozin discontinued (visits 1–2); lisinopril reduced then discontinued; atorvastatin 40 → 20 mg; magnesium and krill oil added
3	Omeprazole and letrozole discontinued; bupropion reduced; magnesium, calcium, vitamin D, and fish oil added
4	Tirzepatide discontinued at enrollment (semaglutide initiated); levothyroxine 88 → 75 mcg; propranolol discontinued; duloxetine switched to sertraline; ondansetron and sumatriptan added; CPAP initiated
5	Topical minoxidil added (visits 5–6)
6	Amitriptyline discontinued (visit 1)
7	Metformin discontinued (visits 1–2); omeprazole discontinued (visits 4–5)

Abbreviations: T2DM, type 2 diabetes mellitus; HTN, hypertension; OSA, obstructive sleep apnea; MAFLD, metabolic-associated fatty liver disease.

**Table 3:** Body composition changes over the 6-month intervention.

Pt	Total Weight Loss (kg)	Fat Mass Loss (kg)	SMM Δ (kg)	% Weight Loss from Fat	BMI Δ (kg/m^2^)	Visceral Fat Change (%)
1	−29.4	−27.2	−1.9	92	−11.8	−67
2 *	−23.3	−24.1	0	100	−7.1	−21
3	−20.7	−17.4	−2.3	84	−7.8	−23
4	−12.0	−14.0	+1.1	100	−4.1	−54
5	−26.8	−22.7	−3.1	85	−10.8	−29
6	−22.8	−20.7	−1.5	91	−2.3	−50
7	−18.1	−16.1	−1.0	89	−6.5	−15
**Mean**	**−21.9**	**−20.3**	**−1.2**	**92**	**−7.2**	**−37.0**

Abbreviations: SMM, skeletal muscle mass; BMI, body mass index. * In Patient 2, fat mass loss (24.1 kg) marginally exceeds total weight loss (23.3 kg), with no net change in skeletal muscle mass. Review of the raw InBody segmental data reveals that total body water increased by 0.6 kg and dry lean mass increased by 0.2 kg between assessments, indicating the patient was better hydrated at the follow-up scan. Because bioelectrical impedance analysis estimates body compartments based in part on tissue water content, this modest hydration difference shifted the impedance-derived partitioning between fat and lean mass, producing the apparent arithmetic inconsistency. Total body weight change remains accurate. This is a recognized artifact of segmental BIA methodology and does not affect the overall interpretation of outcomes.

**Table 4:** Baseline and 6-month body composition (BIA-estimated), shown as baseline → 6 months. Visceral fat level is a unitless index; baseline values were not retrievable for Patient 4.

Patient	Fat Mass (kg)	Skeletal Muscle Mass (kg)	Visceral Fat Level
1	44.6 → 17.4	28.4 → 26.5	21 → 6.9
2	72.8 → 48.7	39.5 → 39.5	29 → 22.9
3	56.8 → 39.4	29.4 → 27.1	26 → 20.0
4	—	—	—
5	63.0 → 40.3	30.3 → 27.2	26 → 18.5
6	43.1 → 22.4	24.1 → 22.6	22 → 11.0
7	55.3 → 39.2	27.8 → 26.8	20 → 17.0

**Table 5:** Changes in metabolic markers and lipid profiles from baseline to 6 months.

Pt	FI Δ (µIU/mL)	A1c Δ (%)	hsCRP Δ (mg/L)	ALT Δ (U/L)	AST Δ (U/L)	TC Δ (mg/dL)	TG Δ (mg/dL)	LDL Δ (mg/dL)	HDL Δ (mg/dL)	VLDL Δ (mg/dL)
1	−23.5	≤0.1	−1.0	−107	−36	−32	−74	−27	+10	—
2	−4.5	−3.1	+3.0	−7	−7	+26	−120	+15	+10.8	0
3	−10.2	−0.7	−6.0	−6	−4	−16	−249	+29	+5.5	−50
4	−10.2	+0.1	−0.8	−4	−3	+83	−41	+73	+18.2	0
5	−10.4	+0.3	−27.4	−4	−2	−7	+7	+10	+1.5	+2
6	−14.8	−0.4	−6.7	+2	+8	−27	−29	−23	+7	−5.6
7	−35.5	−1.8	−10.9	−10	−15	+10	−38	+15	+2.2	−8

Abbreviations: FI, fasting insulin (µIU/mL); A1c, Hemoglobin A1c (%); hsCRP, high-sensitivity C-reactive protein (mg/L); ALT, alanine aminotransferase (U/L); AST, aspartate aminotransferase (U/L); TC, total cholesterol; TG, triglycerides; LDL, low-density lipoprotein; HDL, high-density lipoprotein; VLDL, very low-density lipoprotein (all mg/dL). Δ = change from baseline to 6 months.

## Data Availability

The data supporting the findings of this study are available from the corresponding author upon reasonable request.

## Abbreviations

The following abbreviations are used in this manuscript:

ALT	Alanine aminotransferase.
AST	Aspartate aminotransferase.
BMI	Body mass index.
βHB	β-Hydroxybutyrate.
GLP-1	RA Glucagon-like peptide-1 receptor agonist.
HDL	High-density lipoprotein.
hsCRP	High-sensitivity C-reactive protein.
HTN	Hypertension.
LDL	Low-density lipoprotein.
MAFLD	Metabolic-associated fatty liver disease.
OSA	Obstructive sleep apnea.
SMM	Skeletal muscle mass.
T2DM	Type 2 diabetes mellitus.
TC	Total cholesterol.
TG	Triglycerides.
VLDL	Very low-density lipoprotein.
